# The Municipal Control of Measles

**Published:** 1896-06-06

**Authors:** 


### The Municipal Control of Measles.

The continued loss of life from measles which goes on year after year without any sign of abatement is a matter of no small concern, and one which must before long attract the attention, if not of the Legislature, at least of our local sanitary authorities.

In the thirty-three towns dealt with by the Registrar-General in his weekly returns, the deaths from measles during last year amounted to 5,623, being far ahead of those from any other zymotic disease, unless we include diarrhœa under that designation. Even in London, where diphtheria has become so large a factor in the zymotic death-rate, the loss of life caused by it is far exceeded by that from measles; yet we allow measles to go on absolutely uncontrolled, notwithstanding that it kills more people than diphtheria, and more than three times as many as scarlet fever. On this important subject Dr. Waldo, the energetic medical officer of health for St. George the Martyr, Southwark, has just issued a double manifesto, drawing attention to it not only in his annual report, just issued, but also, along with Dr. Walsh, in an article, entitled "Murder by Measles," in this month's *Nineteenth Century*.

Although many people look upon measles as a comparatively simple affair, the case mortality being but small among the well-to-do, it is a very serious affair indeed among the poor and where there is overcrowding or the sanitary conditions are defective. According to Dr. Parkes, in some of the poor neighbourhoods the case mortality of measles amounts to as much as 20, or even 30, per cent., a death-rate which is comparable only with such diseases as diphtheria or cholera.

The case, then, for State or municipal interference with measles is undoubtedly very strong, overwhelming in fact, if it can be shown that such interference as is within the bounds of practical possibility will do good.

The objection which has from the beginning been raised against making measles notifiable, like other infectious diseases, is that from its infectiveness being so marked a feature even before the malady is capable of being diagnosed, it would be impossible, however soon removal were practised, to stop the spread of the disease. This objection is a very valid one, and it seems to us that Dr. Waldo and those who go with him rather spoil their case by so definitely linking together notification and isolation, especially as even the wealthiest sanitary authorities might well hesitate to provide the immense hospital accommodation which would be necessary for the complete isolation of measles. But we would point out that notification combined with a very moderate amount of hospital accommodation may do much good, quite independently of any attempt to stamp out the disease by isolation.

The first and most obvious way in which notification would do good would be by enabling the authorities to keep out of the schools those who come from infected houses. But there are other ways in which it would be beneficial, especially by giving the sanitary authorities early warning where to go with disinfectants, where to cleanse, and where to urge removal to hospital. The proper function of hospitals is, however, another matter which must be separately considered. Isolation must not be looked upon as the primary or the main use of hospitals for measles.

If they were useful only by virtue of the isolation they afforded, we should not see any prospect of great reward for the outlay which would be involved in providing hospitals for measles. But the primary use of all hospitals is treatment, and in few diseases is treatment likely to be more useful and life-saving than in cases of measles selected from among the poorer quarters of the town. There is but little reason to believe that people in good houses escape measles any more than poorer people, but they die from it far less.

Measles is fatal chiefly by virtue of the complications which occur in its course. These are largely the result of inefficient nursing and insanitary surroundings; and there can be but little doubt that an immense saving of life could be effected if the sanitary authorities had at their backs a moderate amount of hospital accommodation for the treatment of the worst cases and those occurring in the most squalid homes; and if, by notification, they had a complete knowledge of the progress of the disease, so that they could enforce disinfection where it was required. What we would especially emphasise is the fact that considerable control may be exercised over measles by the authorities without going to the enormous expense of attempting any complete system of hospital isolation. It is, doubtless, a grand thing to stop an epidemic, but it is almost as good to stay its fatality.

